# Relevance of assistive technology and the sustainable development goals to stakeholder organizations in Malawi

**DOI:** 10.1080/16549716.2022.2133381

**Published:** 2022-11-09

**Authors:** Emma M. Smith, Ikenna D. Ebuenyi, Juba A. Kafumba, Monica Jamali-Phiri, Alister Munthali, Mac MacLachlan

**Affiliations:** a Assisting Living and Learning Institute, Maynooth University, Maynooth, Ireland; b Centre for Social Research, Olomouc University Social Health Institute (OUSHI), Palacky University, Olomouc, Czech Republic

**Keywords:** Assistive technology, sustainable development goals, global health, health policy, collective leadership

## Abstract

Assistive technologies are critical to supporting the participation and engagement of persons with disabilities and others who experience functional difficulties in daily life. Assistive products have been demonstrated to be related to the achievement of the Sustainable Development Goals (SDGs); however, no previous research has explored the relationship between assistive technology (AT) and the SDGs from the perspective of stakeholder organisations working in the field of AT provision. In this study, we evaluated the relevance of AT and the SDGs to achieving the organisational missions of key stakeholders in AT ecosystem in Malawi. Key stakeholders (n = 36) in the AT field in Malawi were asked to rate the relevance of AT to achieving their organisational missions, and the relevance of AT to each of the 17 SDGs on a 5-point Likert scale. Stakeholders who participated were engaged in consultative meetings with the government and an action research team as part of a larger policy development project, and represented ministries and government agencies, organisations of persons with disabilities, and local and international non-governmental organisations. AT was rated as being relevant to all of the SDGs, albeit to varying degrees, and not surprisingly to achieving AT stakeholders’ organisational missions. The cross-cutting nature of the relevance of AT underscores the importance of cross-ministerial cooperation and shared leadership in provision AT.

## Introduction

Assistive technology (AT) is a crucial component for enabling the participation and inclusion of persons with disabilities, ageing individuals, and others who experience limitations in participation in society [1]. AT is defined by the World Health Organization as the ‘systems and services related to the delivery of assistive products and services,’ while Assistive Products (APs) are defined as the products which ‘maintain or improve an individual’s functioning and independence, thereby promoting their wellbeing.’ [1] These products and systems are core to the Global Cooperation on Assistive Technology (GATE), a WHO initiative which seeks to improve access to AT for all who require it – an estimated 1 billion people worldwide [2].

APs are also central to the realisation of rights enshrined in the UN Convention on the Rights of Persons with Disabilities (CRPD) [3], and to the achievement of the Sustainable Development Goals (SDGs) [4]. The SDGs were established in 2015 as a ‘blueprint to achieve a better and more sustainable future for all.’ [5] These 17 interconnected goals address a range of issues related to global wellness and sustainability, from those intended to reduce poverty and increase access to health and wellness, to climate, peace and justice, and green energy. Notably, they emphasise the inclusion of those from marginalised groups, including persons with disabilities, based on an understanding that inclusion of all members of society is critical to global sustainability.

The relevance of AT to achieving all 17 of the SDGs was demonstrated by Tebbutt et al., in 2016, through a review of the relevant literature. This evidence-based approach linked existing research in AT to the achievement of each of the SDGs [4]. Demonstrating the links between AT and the SDGs has allowed advocates to justify the benefits of AT to national governments, highlighting their role in the achievement of SDG-related targets in relevant policy and strategies [6].

Recently, the World Health Assembly issued resolution WHO71.8 which calls on governments to ‘develop, implement and strengthen policies and programs … to improve access to assistive technology within universal health and/or social services coverage.’ [7] The resolution makes further recommendations in the areas of human resources; access to appropriate technologies; the use of a priority APL; investment in research, development, and innovation; international cooperation; population-based data collection; and investment in barrier-free environments. Each of these recommendations, taken together, promotes inclusion and the achievement of rights for those who use or require APs.

Identifying the relevance of AT to the achievement of the SDGs provides justification for the provision of AT as a strategy to fulfil countries’ commitments to achieving the SDGs. Furthermore, it serves to reinforce an understanding that the impact of AT is broad and goes beyond health and welfare. While this link has been documented in the academic literature, no work has been undertaken to demonstrate the link between AT and the SDGs from the perspective of key stakeholders representing the breadth of AT engagement in a national context. This is important, as these stakeholders play a substantial role in advocating for AT policy and systems. Therefore, the objective of this research was to determine the relevance of AT to the achievement of the SDGs for a range of stakeholders in Malawi. The knowledge from this research will be used to inform AT systems development following the development of relevant AT policy.

## Methods

This research was conducted as a quantitative survey with a purposive sample. Data were collected during a workshop of assistive technology stakeholder organisations in Malawi, which was conducted as part of the broader APPLICABLE Action Research Project [8].

This workshop was to launch the development of a national APL and seek feedback from participants about the nature of this list and an associated policy. Informed consent was sought by all attendees prior to the workshop to use data gathered throughout for research, and the study was approved by the University of Malawi and Maynooth University Social Research Ethics Committee.

Data were collected using a paper-based survey distributed to participants of the APPLICABLE Action Research Group stakeholder consultation (n = 44 workshop participants, n = 36 survey respondents). Participants were invited to the workshop in consultation with government ministries on the basis that they were representatives of organisations working in Malawi in the area of AT. Participants represented government ministries and agencies (n = 10), organisations of persons with disabilities (n = 3), health service providers (i.e. hospitals; n = 7), schools (n = 2), local non-governmental organisations (n = 7), international non-governmental organisations (n = 13), and one independent consultant. Data were collected following a presentation about AT and the World Health Organization Assistive Product List, in which a definition of AT and APs was provided, as well as a review of the SDGs. Completion of the survey was voluntary and anonymous. Thirty-six of a total 44 attendees completed the survey. For each of the 17 SDGs, respondents were asked to rate two questions on a 5-point Likert scale (0–4) for each of the 17 SDGs: 1) To what extent is AT relevant to the achievement of the SDG? and 2) How relevant is this SDG to your organisation’s mission? Likert ratings were defined as follows: 0 – No relevance; 1 – Very Low Relevance; 2 – Low Relevance; 3 – High Relevance; 4 – Very High Relevance. To evaluate the relevance of the work of AT-related organisations to the SDGs, mean scores for each SDG were calculated for each of the relevance scores. To calculate relative relevance, the product of the two relevance scores was calculated by respondent, and the mean of the product scores across all organisations is reported in the table.

## Results

**Figure 1.**
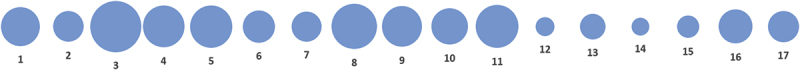
Relative Importance of the Sustainable Development Goals to AT Organisations.

A total of 36 stakeholders from organisations working in the area of AT provided responses to the survey. The mean scores for each of the SDGs for each of the questions, and the mean product of the two questions are provided in Table 1. We have also provided the mode of each question to demonstrate the most frequently selected likert value for each SDG. Figure 1 shows the relative relevance across all 17 SDGs. Higher relative relevance indicates higher importance of AT to both the organisation and the achievement of the SDGs. In Figure 1, the size of the bubble is proportional to the relevance product scores; larger bubbles have proportionately higher relevance product than smaller bubbles.

**Table 1.** Relevance of AT and Organisational Mission to achieving the SDGs.

	Relevance of AT to Org		Relevance of SDG to Org		Relative Relevance
Sustainable Development Goal	Mean (SD)	Mode	Mean (SD)	Mode	Mean (SD)
1: No poverty	*3.03 (1.11)*	4	3.22 (0.90)	5	9.97 (5.07)
2: Zero hunger	2.36 (1.36)	2	2.69 (1.35)	4	7.81 (6.21)
3: Good health and well-being	3.50 (1.06)	5	3.44 (1.08)	5	13.14 (5.11)
4: Quality education	2.94 (1.33)	5	3.36 (0.96)	5	10.67 (5.92)
5: Gender equality	3.11 (1.12)	5	3.19 (1.09)	5	10.83 (5.84)
6: Clean water and sanitation	2.67 (1.29)	5	2.81 (1.14)	5	8.28 (5.82)
7: Affordable and clean energy	2.28 (1.41)	3	2.69 (1.41)	5	7.64 (6.01)
8: Decent work and economic growth	3.00 (1.43)	5	3.50 (0.97)	5	11.58 (6.26)
9: Industry, innovation, and infrastructure	3.19 (1.06)	5	3.22 (1.38)	5	10.36 (5.73)
10: Reduced inequalities	2.53 (1.42)	5	3.00 (1.41)	5	9.31 (6.22)
11: Sustainable cities and communities	2.64 (1.20)	5	3.44 (2.81)	5	11.00 (12.49)
12: Responsible consumption and production	1.47 (1.58)	1	2.08 (1.57)	5	4.86 (6.34)
13: Climate action	2.22 (1.55)	5	2.31 (1.51)	3	6.58 (5.88)
14: Life below water	1.72 (1.45)	2	1.92 (1.27)	2	4.53 (5.03)
15: Life on land	1.89 (1.82)	1	1.61 (1.74)	1	5.75 (6.89)
16: Peace, justice, and strong institutions	2.53 (1.58)	5	2.69 (1.51)	5	8.67 (6.35)
17: Partnership for the goals	2.39 (1.36)	4	2.72 (1.43)	5	8.00 (5.46)

AT was identified as being relevant to each of the SDGs, with the highest mean scores for goals #3 (Good health and well-being), #9 (Industry, innovation, and infrastructure), and #5 (Gender equality). Each of the SDGs was also deemed relevant to the organisational missions of at least some of the stakeholders present. The SDGs with relevance across the greatest number of organisations included #8 (Decent work and economic growth), #3 (Good health and well-being), and #11 (Sustainable cities and communities).

## Discussion

This study provides a perspective of the importance of AT to organisations’ achievement of the SDGs. AT was deemed relevant to the achievement of each of the SDGs, which is consistent with the work done by Tebbutt et al., which suggested equitable access to APs is critical to the achievement of all 17 SDGs [4] in a way that ensures nobody is ‘left behind’. AT provision should therefore be understood as a key component of work aimed towards achieving the SDGs. Article 32 of the UNCRPD Article is on international cooperation and is especially concerned with international development assistance. It requires that ‘international development programmes [are] inclusive of and accessible to persons with disabilities’ (A. 32a) and this is ‘including facilitating access to and sharing of accessible and assistive technologies, and through the transfer of technologies’ (A. 32d) [3]. We recommend that governments and development partners ensure AT is addressed across their full range of their projects.

Notably, each of the SDGs was deemed relevant to at least one and often many more of the organisational missions of AT stakeholders in Malawi. AT stakeholders often undertake a breadth of work which includes, but is not limited to, AT specifically. The breadth of stakeholders, and their respective organisational missions, demonstrates the interconnected nature of each of the SDGs. Further exploration of the link between AT and SDGs within the context of organisational missions of key stakeholders should be done in in-depth qualitative work.

The breadth of the SDGs and the potential for AT to impact their achievement underscore the necessity of a collaborative approach across ministries and stakeholders to help achieve these goals. Shared leadership approaches provide the necessary commitment and buy-in from each of the relevant ministries, which may be less apparent or present in a siloed single ministry leadership approach [9]. While AT is often seen as the concern of government departments of health, welfare, or equity, our results demonstrate that, among a sample of key stakeholders in AT in Malawi, AT is seen as relevant to achieving all SDGs. This has important implications for policy and implementation of policy on AT provision. To achieve the broad aims set forth in WHA71.8 and recognise the breadth of areas which are impacted by AT, governments must take an approach that promotes inter-ministerial co-operation.

Our research does not claim to be representative of all AT stakeholders in Malawi, or elsewhere. However, our participants in this study do reflect those stakeholders whom the government felt were important partners to invite to a workshop on AT policy development and implementation. As such, they may be seen as the key stakeholders whose views it is necessary to understand if we wish to enhance AT provision at a national level. All stakeholders who were engaged in this research shared a commitment to the promotion of a just and inclusive society, and to the achievement of WHA71.8. This is consistent with our previously completed network analysis which demonstrates linkages between stakeholders at every level in Malawi [10]. The literature supports this, acknowledging that the complexity of AT provision necessitates an intersectoral and interdisciplinary systemic approach [11].

## Conclusion

Achievement of each of the SDGs is relevant to the work of organisations working in the area of AT. Furthermore, these organisations explicitly acknowledge the importance of AT in contributing to their goals. The interconnected nature of the SDGs, and the relevance of AT to their achievement, underscores the importance of shared or collective leadership in AT policy and systems.

## Abbreviations

AT: Assistive Technology
AP: Assistive Products
CRPD: Convention on the Rights of Persons with Disabilities
WHO: World Health Organization
GATE: Global Cooperation on Assistive Technology
SDGs: Sustainable Development Goals

## Paper context

This paper forms a part of the APPLICABLE project, a participatory research project conducted jointly with the Assisting Living and Learning Institute at Maynooth University and the Centre for Social Research at the University of Malawi. The APPLICABLE project aimed to develop a Priority Assistive Product List and relevant policy in the area of assistive technology. Data gathered for this manuscript can be retrieved by contacting the authors.
